# Gut microbiota and derived metabolomic profiling in glaucoma with progressive neurodegeneration

**DOI:** 10.3389/fcimb.2022.968992

**Published:** 2022-08-12

**Authors:** Yinglei Zhang, Xujiao Zhou, Yi Lu

**Affiliations:** ^1^ Department of Ophthalmology, Eye and ENT Hospital, Fudan University, Shanghai, China; ^2^ Eye Institute, Eye and ENT Hospital of Fudan University, Shanghai, China; ^3^ Key Laboratory of Myopia, Ministry of Health, Shanghai, China; ^4^ Shanghai Key Laboratory of Visual Impairment and Restoration, Shanghai, China

**Keywords:** Glaucoma, retinal ganglion cell (RGC), gut microbiota, microbial metabolite, glutathione

## Abstract

Glaucoma is a multifactorial, neurodegenerative disorder characterized by the loss of retinal ganglion cells (RGCs). Crosstalk between the gut microbiota and host is involved in the progression of many neurodegenerative diseases, although little is known about its role in glaucoma. To investigated the alterations of the gut microbiota and derived metabolites in glaucomatous rats, and the interaction with RGCs, we performed 16S rRNA (V1-V9) sequencing and untargeted metabolomic analyses. The microbial composition differed significantly between the two groups, and the diversity of cecal bacteria was dramatically reduced in glaucomatous rats. The Firmicutes/Bacteroidetes (F/B) ratio, Verrucomicrobia phylum, and some bacterial genera (Romboutsia, Akkermansia, and Bacteroides) were dramatically increased in the glaucomatous rat model compared with the control, which showed negative correlation with RGCs. Untargeted metabolomic analysis identified 284 differentially expressed metabolites, and Kyoto Encyclopedia of Genes and Genomes pathway enrichment analysis revealed considerable enrichment mainly in bile secretion pathways. The relationships among the metabolites enriched in the bile secretion pathway, differentially expressed cecal microbiota, and RGCs were investigated, and glutathione (GSH) was found to be negatively correlated with Bacteroides and F/B and positively correlated with RGCs. Reduced GSH level in the blood of glaucoma rats is further established, and was negatively correlated with Romboutsia and the F/B ratio and positively correlated with RGCs. This finding suggests the potential role of the gut microbiota and derived metabolites in glaucoma, and GSH, a major antioxidant metabolite, was related to their effects, indicating the potential for the development of gut microbiota-targeted interventions for glaucoma.

## Introduction

The gastrointestinal tract constitutes 95% of the human microbiome, and its massive complexity is necessary to properly maintain homeostasis. (León and Francino, 2022) In recent years, a growing body of data is pointing the possible role of gut microbiota dysbiosis in the onset and exacerbation of ocular disease, and eye-gut axis concept is raised. (Floyd and Grant, 2020) The complexity of the gut microbiome is a critical component of this axis, but limited evidence has pointed out the mediators communicating gut microbiota and ophthalmic pathological changes.

Glaucoma is a neurodegenerative disease characterized by loss of retinal ganglion cells (RGCs), which has a multifactorial cause. The mechanisms causing glaucomatous neurodegeneration are not fully understood. Pathological changes in glaucoma include loss of specific nerves and deposition of protein aggregates in particular anatomical areas, and similar biological mechanisms have been suggested for Alzheimer’s disease and Parkinson’s disease. The brain–gut axis has been well studied in these diseases, and it provides a bidirectional communication system between the central nervous system and the gastrointestinal tract *via* diverse signaling pathways, including energy metabolism, oxidative stress, mitochondrial function, and neuroinflammation. (Heintz-Buschart et al., 2018; Das De et al., 2022; Giridharan et al., 2022; Terkelsen et al., 2022) Given the similarities in the immune and neurodegenerative factors between glaucoma and Alzheimer’s disease and Parkinson’s disease, we speculate that microbial communication between the gastrointestinal tract and the eye is involved in the progressive neurodegeneration in glaucoma.

Gong et al. (Gong et al., 2020) found differences in gut microbial contents between POAG patients and controls. In mouse models, glaucomatous neural degeneration was absent under germ-free conditions, whereas progressive RGC loss was observed under specific-pathogen-free conditions. (Chen et al., 2018) These studies support the involvement of gut microbiota alteration in glaucoma; however, additional work is required to elucidate this association and to determine whether gut microbiota have a role in glaucoma progression. In addition, intestinal flora-derived metabolites are the primary mediators in interactions between gut microbiota and the host. Metabolomic studies have identified specific metabolic phenotypes in blood samples and intraocular fluid. (Gong et al., 2020; Wang et al., 2020; Tang et al., 2022) For example, POAG patients had lower amounts of spermine and spermidine polyamines than non-POAG patients in plasma and aqueous humor metabolomic profiles. (Buisset et al., 2019) However, few studies have explored the fecal microbial composition and the resulting metabolic phenotypes in glaucoma. It is necessary to define the aberrations in the gut microbiota and metabolic phenotype to understand how these enhance or inhibit glaucoma progression.

A previous study involving 1999 African American people (1217 with primary open-angle glaucoma (POAG) and 782 controls) found that variants in two mitochondrial DNA (mtDNA) haplogroups, L2a1 (m.15784T>C) and L2 (m.16390G>A), were enriched in the DNA pools of POAG patients compared with the controls, and were associated with the composition of the gut microbiota. (Ma et al., 2014; Collins et al., 2016) Increased mtDNA deletion is paralleled by a decrease in the number of mitochondria per cell and by cell loss in POAG. (Izzotti et al., 2010) POAG patients exhibit a spectrum of mitochondrial abnormalities and glaucoma-related apoptosis could be triggered by oxidative stress *via* mitochondrial damage. (Abu-Amero et al., 2006) As a pivotal antioxidant, the reduction of glutathione might be possible source of oxidative stress. (Ulusu et al., 2003) However, few studies have investigated the role of oxidative stress in the eye-gut axis.

To identify the role of gut microbiota and metabolites in the progression of glaucoma, we compare the gut microbiota composition and the fecal metabolic phenotype in a glaucomatous rat model and control, using 16S rRNA sequencing and liquid chromatography-mass spectrometry metabolomics. The interaction of RGC loss with the cecal microbiota and the derived metabolites is also investigated to provide insights into potential disease pathways and pave the way for the development of neuroprotective interventions.

## Materials and methods

### Animals

The animal experiments were approved by the Ethics Committee for Animal Studies at the Eye & ENT Hospital of Fudan University, Shanghai, China, and experimental procedures conformed to the Association for Research in Vision and Ophthalmology statement for the use of animals in research. Experiments were performed in 2-month-old male Wistar rats (150–200 g, SLAC Laboratory Animal Co., Ltd., Shanghai, China). All animals were maintained in cages with a 12 h light–dark cycle environment at 22 ± 2°C and were given a regular diet for 35 days.

### Chronic glaucoma model in rats

Male Wistar rats were randomly divided into glaucomatous and control groups. All rats were anesthetized by intraperitoneal injection of 10% chloral hydrate (3.6 mL/kg). Oxybuprocaine hydrochloride eye drops (0.4%, Santen Pharmaceutical Co., Ltd., Osaka, Japan) were administered as a topical anesthetic. In the glaucomatous group, three episcleral veins located near the superior and inferior rectus muscles of bilateral eyes were isolated and cauterized precisely. In the control group, bilateral eyes underwent a sham operation, which involved isolating the veins without cauterization. Intraocular pressure (IOP) was measured with a calibrated tonometer (Tono-Pen XL, Reichert, Depew, NY, USA) before treatment and at 1, 3, 5, 7, 14, 21, 28, and 35 days after treatment. One randomly selected eye was measured in each rat and was recorded as the mean of five consecutive measurements with a deviation of <5%.

### Retrograde labeling and counting of RGCs

Thirty-five days after episcleral vein cauterization, anesthetized rats were immobilized in a stereotactic apparatus and the superior colliculi were microinjected bilaterally with FluoroGold (3%, 2.0 µL, Sigma-Aldrich, St. Louis, MO, USA), as previously described. (Ju et al., 2005) Seven days after fluorescent tracer injection, labeled retinas were dissected and flat-mounted on glass slides with the ganglion cell layer facing up. Images were captured with a fluorescence microscopy (DM4000 B, Leica, Germany) at 20× magnification and the cells were counted. (Zhou et al., 2017)

### Sample collection and DNA extraction

At the baseline and after 35 days, the cecal contents of the rats were collected in sterile tubes and stored at −80°C until microbial analysis. The DNA was extracted from 200 mg cecal samples using the CTAB method. DNA concentration and purity were then monitored by running the samples on 1.0% agarose gels.

### 16S rRNA gene sequencing of the gut microbiota and bioinformatic analysis

We performed a full-length sequence with all variable regions (V1-V9), which provides better taxonomic resolution and is possible to classify nearly all sequences as the correct species, while sub-regions may show bias in the bacterial taxa. (Johnson et al., 2019) Polymerase chain reaction (PCR) amplification was performed with barcoded primers (8F 5′-AGAGTTTGATCCTGGCTCAG-3′ and 1509R 5′-GNTACCTTGTTACGACTT-3′). All PCR reactions were carried out with TransStart FastPfu DNA Polymerase (TransGen Biotech, Beijing, China). After mixing the PCR products in equidensity ratios, the mixture was purified with a QIAquick@ Gel Extraction Kit (Qiagen, Hilden, Germany). Sequencing libraries were generated using a SMRTbell Template Prep Kit (PacBio, Menlo Park, CA, USA) following the manufacturer’s recommendations, and the library quality was assessed on a fluorometer (Qubit 2.0, Thermo-Fisher Scientific, Waltham, MA, USA). The library was sent for sequencing on the PacBio Sequel platform (PacBio), which was conducted by Novogene Bioinformatics Technology Co., Ltd. (Beijing, China).

Raw sequences were initially processed through the PacBio SMRT portal (PacBio). Sequences were filtered for a minimum of three passes, and a minimum predicted accuracy of 90% (minfullpass = 3, minPredictedAccuacy = 0.9). The files generated by the PacBio platform were then used for amplicon size trimming to remove sequences outside the expected amplicon size (minimum length 1340 bp, maximum length 1640 bp). The reads were assigned to samples based on their unique barcode and truncated by cutting off the barcode and primer sequence. After removing chimera sequences, the clean reads were obtained.

Sequence analysis was performed with UPARSE software (version v7.0.1001, http://drive5.com/uparse). Sequences with ≥97% similarity were assigned to the same operational taxonomic units (OTUs). The OTU abundance information was normalized using a sequence number standard corresponding to the sample with the fewest sequences. Beta diversity metrics were calculated with QIIME (version 1.9.1). (Caporaso et al., 2010) Principal coordinate analysis was performed to obtain principal coordinates and visualize complex, multidimensional data. A matrix of unweighted UniFrac distances among samples obtained before was transformed to a new set of orthogonal axes, in which the first maximum variation factor was shown by the first principal coordinate, the second was shown by the second principal coordinate, and so on. Alpha diversity (Shannon index and Chao 1 index) in our samples was calculated with QIIME (version1.9.1) and displayed with R software (version 2.15.3).

### Untargeted metabolomic analyses

The gut metabolomic profiles of the rats were measured from stool samples using ultra-high performance liquid chromatography-tandem mass spectrometry (UHPLC-MS/MS), which was performed with a UHPLC system (Vanquish, Thermo-Fisher Scientific) coupled with a high-field mass spectrometer (Orbitrap Q Exactive, Thermo-Fisher Scientific) at Novogene Co., Ltd. (Beijing, China). Samples were injected onto a Hypesil Gold column (100 × 2.1 mm, 1.9 μm) at a flow rate of 0.2 mL/min. The eluents for the positive polarity mode were 0.1% formic acid in water (eluent A) and methanol (eluent B). The eluents for the negative polarity mode were 5 mM ammonium acetate (pH 9.0, eluent A) and methanol (eluent B). The mass spectrometer was operated in positive/negative polarity mode with spray voltage of 3.5 kV, capillary temperature of 320°C, sheath gas flow rate of 35 arb, auxiliary gas flow rate of 10 arb, S-lens RF level of 60, and auxiliary gas heater temperature of 350°C.

The metabolites were annotated using the Kyoto Encyclopedia of Genes and Genomes (KEGG) database (https://www.genome.jp/kegg/pathway.html), the HMDB database (https://hmdb.ca/metabolites), and the LIPIDMaps database (http://www.lipidmaps.org/). Partial least squares-discriminant analysis (PLS–DA) was performed with metaX. (Wen et al., 2017) The metabolites with variable importance in the projection (VIP) of >1, P-value of <0.05, and fold change of ≥1.5 or ≤0.667 were considered differential metabolites. Volcano plots were used to filter metabolites of interest based on log2(fold change) and −log10(P-value) of metabolites by ggplot2 in R language.

The functions of these metabolites and metabolic pathways were studied using the KEGG database. The metabolic pathway enrichment of differential metabolites was performed. When the ratio was satisfied by x/n > y/N, the metabolic pathway was considered enriched, and when the P-value of the metabolic pathway was <0.05, the metabolic pathway enrichment was statistically significant.

### Determination of endogenous glutathione in rat blood

To avoid variations and glutathione (GSH) loss, we focused on blood collection, initial processing, and storage. Blood samples were collected at the same time in tubes prefilled with 23 μL of tripotassium EDTA and 100 μL of N-ethylmaleimide per milliliter of blood, as described previously. (Giustarini et al., 2013) A UHPLC–MS/MS system (Santa Clara, CA, United States) in multiple-reaction monitoring mode was used to quantitate the GSH level. The standard solution and samples were injected into a Waters XSelect HSS T3 column (2.1 × 100 mm, 1.7 μm), which was kept at 45°C. The mobile phase, consisting of water with 20mM ammonium acetate (solvent A) and 90% acetonitrile (solvent B), was delivered at a flow rate of 0.25 mL/min. The solvent gradient was as follows: initial 92% B, 9 min; 72% B, 9.5–11.5 min; 50% B, 12–15 min; 92% B. The mass spectrometer was operated in negative multiple-reaction monitoring mode. Parameters were as follows: ion spray voltage, 4000 V; curtain gas, 45 psi; gas flow, 5 L/min; ion source temperature, 300°C; and sheath gas flow, 11 L/min. In addition, hemoglobin (Hb) concentration in the treated blood samples was measured according to the manufacturer’s instructions for Drabkin’s reagent (Sigma-Aldrich). GSH values in rat blood were expressed in units of nanomoles per milligram Hb.

### Statistical analysis

Statistical analyses were performed using SPSS (version 22.0, IBM, Armonk, NY, USA). All data are presented as mean ± standard deviation. Normally distributed data were compared and analyzed using a two-tailed Student’s t-test, and non-normal distribution data were compared between the two groups using the Mann–Whitney test and analysis of similarities (ANOSIM). The comparison of IOP at different time between the two groups was performed using two-way repeated measures ANOVA. Spearman’s rank correlation was conducted to measure the correlation of two continuous variables. Receiver-operating characteristic (ROC) curve analysis and calculation of the area under the ROC curves (AUC) were used to compare the diagnostic performance of candidate metabolites for discrimination between the glaucomatous and control groups. P-values of <0.05 were considered statistically significant, and P-values were corrected for multiple comparisons by the Benjamini–Hochberg false discovery rate (FDR) using R software (version 2.15.3).

## Results

### IOP measurements and RGC loss in the glaucomatous and control groups

**Figure 1:**
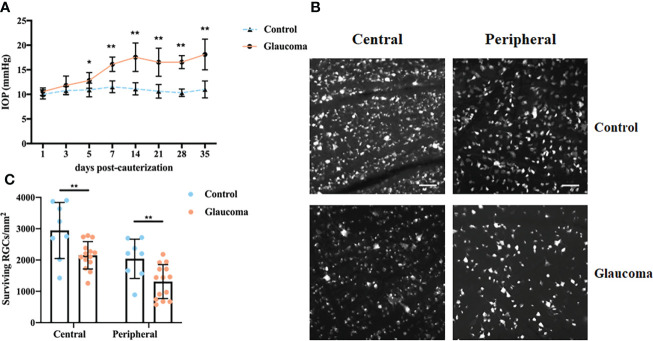
IOP changes and FluoroGold labeling of RGCs in control and glaucomatous rats. **(A)** Changes in IOP after inducing chronic ocular hypertension through EVC. **(B)** FluoroGold labeling of surviving RGCs in retinas 5 weeks after EVC. Images (scale bar, 50μm) from the same retina in central and peripheral parts are showed. **(C)** Quantitative analysis of RGCs in control and glaucomatous eyes. The results are expressed as the mean ± SD. *P<0.05, **P<0.001. IOP, intraocular hypertension; RGC, retinal ganglion cell; EVC, episcleral vein cauterization; SD, standard deviation.

Ocular hypertension was induced in Wistar rats *via* episcleral vein cauterization. IOP was significantly elevated at 5 days after cauterization compared with the eyes in the control group (12.83 ± 1.60 mmHg versus 10.90 ± 1.38 mmHg respectively, *P = 0.012, Two-way repeated measures ANOVA*, **Figure 1**). The elevation was observed at all subsequent measurement points (all P < 0.001, Two-way repeated measures ANOVA, **Figure 1A**). To establish the RGC loss in glaucomatous rats 35 days after the induction of ocular hypertension, the number of FluoroGold-labeled RGCs was counted (**Figures 1B, C**). Compared with the control group, central and peripheral RGCs had decreased significantly in the glaucomatous group (both P < 0.001, Student’s t-test, **Figure 1B**). In addition, there was no significant difference in the body weight of rats between the control and glaucomatous rats (284.3g ± 13.6g versus 278.4g ± 16.1g, P = 0.634, Student’s t-test).

### Structure of the gut microbiome and correlation with RGCs

**Figure 2:**
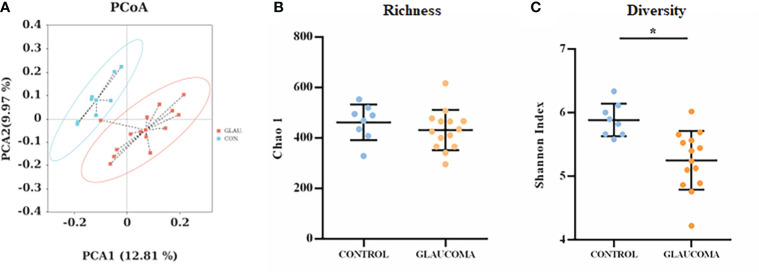
Diversity and richness of the gut microbiota in rats. **(A)** PCoA score plots based on unweighted UniFrac distance metrics (β-diversity) in the glaucomatous and control rats. α-diversity at the OTU levels indicated by the Chao 1 index **(B)** and Shannon index **(C)** are compared between glaucoma and control groups. *P<0.05. OTU, operational taxonomic unit; PCoA, principal coordinate analysis.

To determine the effect of ocular hypertension on the gut microbiota, we profiled the gut microbial community composition using 16S rRNA gene sequencing. Analysis of the two groups using the shared and exclusive OTUs in fecal samples was plotted as a Venn diagram (**Supplementary Figure S1**). Principal coordinate analysis (PCoA) was performed to investigate the β-diversity of the microbial community in the glaucomatous and control groups based on unweighted UniFrac distance metrics (**Figure 2A**). The plot results demonstrated that the microbial composition differed significantly between the two groups (ANOSIM, R = 0.213, P = 0.016). No significant difference was found in the Chao 1 index between the two groups, indicating similar richness at the OTU level (P = 0.365, Student’s t-test, **Figure 2B**). The Shannon index, which considered the numbers of OTUs and their relative abundance, was significantly lower in the glaucomatous group, suggesting less diverse microbial communities compared with the control rats (P = 0.034, Student’s t-test, **Figure 2C**).

**Figure 3:**
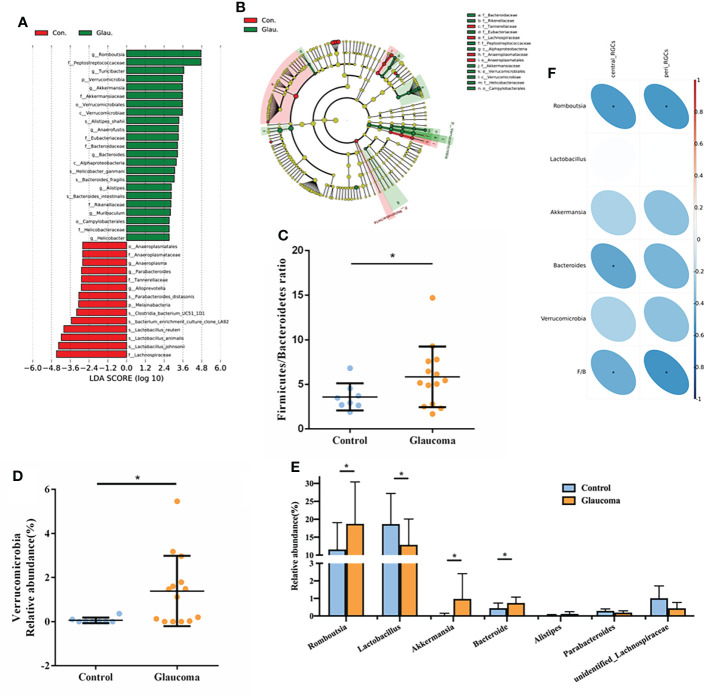
Detailed analysis of microbial abundance and its correlation with RGCs. **(A)** LDA scores (log10) of the bacterial taxa differentially abundant between glaucoma and control rats using LEfSe analysis (LDA > 2.0). Red bars indicate taxonomic enrichment in control rats, and green bars indicate taxonomic enrichment in glaucomatous rats. **(B)** Cladograms generated by LEfSe analysis indicate different taxonomic levels between control and glaucoma groups. Red circle indicates taxonomic enrichment in control rats, and green circle indicate taxonomic enrichment in glaucomatous rats. **(C)** Comparison of the Firmicutes/Bacteroidetes ratio in glaucoma and control groups. *P<0.05. **(D)** Relative abundance of the Verrucomicrobia phylum in glaucoma and control groups. *FDR-corrected q<0.05 **(E)** Relative abundance of the 7 genera differentially enriched in glaucoma and control groups (all P<0.05, *FDR-corrected q<0.05). **(F)** Spearman’s correlation analysis is done and heatmap shows the correlation between differential bacteria and central/peripheral RGCs. Correlation coefficient is on the right side, and red indicates positive correlation and blue indicates negative correlation. RGC, retinal ganglion cell; LDA, linear discriminant analysis; LEfSe, linear discriminant analysis effect size; FDR, false discovery rate.

To determine the relative abundance of the dominant bacteria, linear discriminant analysis (LDA) scores and linear discriminant analysis effect size (LEfSe) analysis were used. The LDA distribution diagram (LDA > 2) showed a clear alteration of the microbial community composition in glaucomatous rats compared with the control rats (**Figures 3A, B**). To confirm the differences in bacterial abundance between the glaucomatous and control groups, standard statistical analysis (Mann–Whitney test) was performed at the phylum and genus levels on the relative abundance of each taxon that was identified by LEfSe as being differentially abundant.

At the phylum level, the most abundant taxon detected in the cecal microbiota of rats was Firmicutes (73.3%), followed by Bacteroidetes (20.3%). Compared with the control, we found a significantly higher Firmicutes/Bacteroidetes (F/B) ratio in the glaucoma group (P = 0.028, **Figure 3C**). The glaucoma group was characterized by a significantly higher Verrucomicrobia level compared with the control group (FDR-corrected q = 0.046, **Figure 3D**). Consistent with the LEfSe analysis results, there were no significant differences in the relative abundance of other phyla between the glaucomatous and control rats (all FDR-corrected q > 0.1). At the genus level, the Romboutsia (FDR-corrected q = 0.047, **Figure 3E**), Akkermansia (FDR-corrected q = 0.011, **Figure 3E**), and Bacteroides (FDR-corrected q = 0.008, **Figure 3E**) levels were significantly higher in the glaucomatous group, whereas the Lactobacillus level was more abundant in the control rats (FDR-corrected q = 0.037, **Figure 3E**).

The correlations between microbial components and the number of central and peripheral RGCs was investigated (**Figure 3F**). The number of central RGCs was negatively correlated with Romboutsia (rho = −0.519, P = 0.013), Bacteroides (rho = −0.469, P = 0.028), and the F/B value (rho = −0.432, P = 0.045). In addition, the number of peripheral RGCs was negatively correlated with Romboutsia (rho = −0.524, P = 0.012) and the F/B ratio (rho = −0.540, P = 0.009).

### Cecal metabolomic profiling in glaucoma and control rats

**Figure 4:**
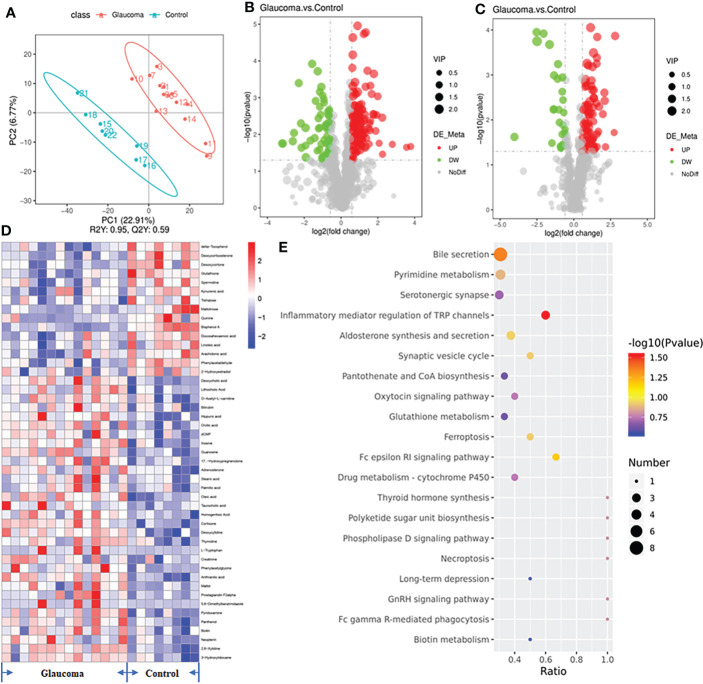
Untargeted metabolomics of the microbiota-derived metabolome. **(A)** The clustering analyses of partial least-squares discriminant analysis (PLS-DA). **(B)** The volcano plot of differential metabolites in positive polarity mode. **(C)** The volcano plot of differential metabolites in negative polarity mode. Grey circle represents a nonsignificant difference, red circle represents an upregulation in metabolites, and green represents a downregulation in metabolites. **(D)** Heatmap of the 47 significantly different metabolites involved in KEGG pathways across glaucomatous and control rats. **(E)** Diagram of the KEGG pathways (top 20). The size of the points represents the number of differential metabolites in the pathway, and the color of the point represents the P value of the hypergeometric test. KEGG, Kyoto Encyclopedia of Genes and Genomes.

We explored the gastrointestinal metabolome in the glaucoma group and control group using an untargeted approach to identify the metabolic pathway of the intestinal microbiome that was involved in glaucoma. We identified a total of 1044 compounds in positive mode and 546 compounds in negative mode. The results of PLS–DA of the samples showed a distinct clustering of rats in the glaucomatous and control groups, indicating that RGC impairment changed the composition of the cecal metabolome (**Figure 4A**, R2 = 0.95, Q2 = 0.59). We identified 284 metabolites in the cecal contents that were differentially expressed between the glaucomatous and control groups, including 203 metabolites that were upregulated and 81 metabolites that were downregulated in the glaucomatous group relative to the control group (**Figures 4B, C**). To demonstrate the biological functions of the differentially expressed metabolites, we performed KEGG pathway enrichment analysis and 47 metabolites involved in KEGG pathways expressed differentially between the two groups (**Figure 4D**, **Supplementary Table S1**). The differentially expressed metabolites in the cecal contents of the glaucomatous and control rats were significantly enriched in bile secretion (N = 8) and inflammatory mediator regulation of TRP channels (N = 4, P = 0.044, P = 0.029, respectively, **Figure 4E**).

**Figure 5:**
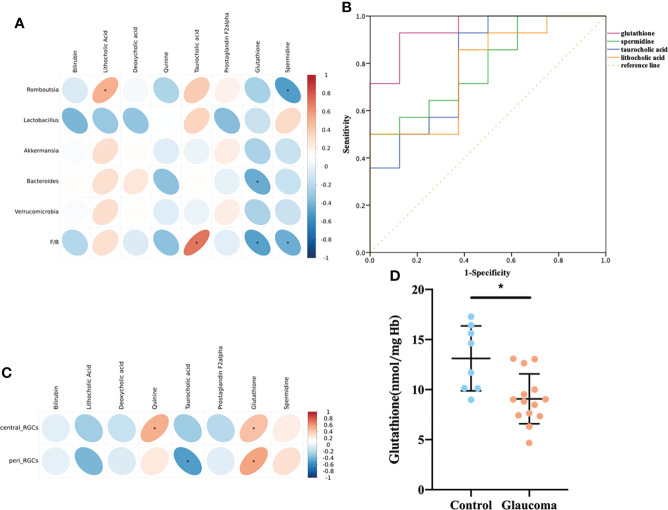
Relationship among the metabolites enriched in the bile secretion pathway, differentially expressed cecal microbiota and RGCs. **(A)** Correlation between differential bacteria and metabolites involved in the bile secretion pathway. **(B)** Receiver-operating characteristic (ROC) curve analysis of metabolites. The area under the curve (AUC) values close to 1 indicates that a high TPR is achieved with low FPR. **(C)** Correlation between differential metabolites involved in the bile secretion pathway and central/peripheral RGCs. **(A, C)** Correlation coefficient is on the right side, and red indicates positive correlation and blue indicates negative correlation. **(D)** Comparison of the glutathione in blood between glaucoma and control groups. *P<0.05. RGC, retinal ganglion cell; TPR, true positive rate; FPR, false positive rate.

The correlations among the eight metabolites that were enriched in the bile secretion pathway and differentially expressed in the cecal contents was investigated by Spearman’s correlation analyses (**Figure 5A**). Lithocholic acid was positively correlated with Romboutsia (P = 0.016), and taurocholic acid was positively correlated with the F/B value (P < 0.001). GSH was negatively correlated with Bacteroides and the F/B value (P = 0.039, P = 0.020, respectively), and spermidine was negatively correlated with Romboutsia and the F/B value (P = 0.017, P = 0.045, respectively). In addition, the results of ROC analysis of the metabolites of lithocholic acid, taurocholic acid, GSH, and spermidine and their correlation with disease yielded AUC values of 0.78, 0.80, 0.95, and 0.79, respectively, indicating that the prediction of glaucoma was reliable (**Figure 5B**, all P < 0.05).

In addition, the correlation between the eight metabolites and RGCs was also investigated (**Figure 5C**). Central RGCs were positively correlated with Quinine (rho=0.510, P=0.015), and peripheral RGCs were negatively correlated with Taurocholic acid (rho=-0.504, P=0.017). Central and peripheral RGCs were both positively correlated with GSH (rho = 0.430, P = 0.046; rho=0.555, P=0.007).

### Blood GSH measurement

According to the above results, cecal GSH was significantly correlated with microbial level and the central/peripheral RGCs (**Figures 5A, C**). To further establish the role of GSH, we quantified GSH in rat blood. The level of blood GSH was significantly decreased in glaucomatous rats (P = 0.004, **Figure 5D**) compared with the controls. The correlations between GSH and cecal microbiota and the numbers of central and peripheral RGCs were analyzed further. The GSH level was negatively correlated with Romboutsia (rho = −0.441, P = 0.040) and the F/B ratio (rho = −0.625, P = 0.002) and positively correlated with numbers of central RGCs (rho = 0.538, P = 0.010) and peripheral RGCs (rho = 0.403, P = 0.043).

## Discussion

A healthy gut microbiome is characterized by a rich and diverse ecosystem, which is presumed to reflect stability and resilience. Alterations to the gut microbiota have been implicated in the pathogenesis of many acute and chronic diseases. (Cho and Blaser, 2012; Lozupone et al., 2012) However, relatively few studies have focused on the gut–eye axis. (Zinkernagel et al., 2017; Gong et al., 2020; Moon et al., 2020) In the current study, we performed 16S rRNA sequencing and untargeted metabolomic analysis, and we found a significant decrease in microbial diversity and different bacterial populations in rats with chronic glaucoma compared with the controls, as well as a distinct alteration of gut metabolites. There were significant correlations between microbial/metabolomic levels and RGCs. Specifically, the microbiota-associated metabolite GSH was positively associated with central/peripheral RGCs, which may provide a new insight into the pathogenesis of glaucoma.

First, we found that the diversity of the gut microbial community decreased in glaucomatous rats. Shannon diversity is strongly associated with gut physiology, and low-diversity, disease-associated microbiomes have been identified in various diseases. (Duvallet et al., 2017) Similarly, a remarkably reduction in the bacterial diversity of the fecal microbiota is also observed in Alzheimer’s disease and Parkinson’s disease. The low diversity of the gut microbial community may disrupt microbiota functions, which are essential for maintaining health or may introduce processes that promote disease. (Elfil et al., 2020; Ling et al., 2020) Reduced microbial diversity in rat model may have an implication for the involvement of cecal microbiota in glaucoma.

We confirmed that Verrucomicrobia and F/B ratio are dramatically increased in the glaucomatous group at the phylum level. Verrucomicrobia is a phylum of gram-negative bacteria that has shown a positive correlation with the forkhead box protein P3 (Foxp3) gene, which expresses anti-inflammatory and immunity in humans and causes microglia activation and differentiation into macrophages at the optic nerve, ultimately damaging ganglion cells. (Bergmann et al., 2011; Fujio-Vejar et al., 2017; Lindenberg et al., 2019; Kuhn et al., 2021) Gram-negative bacterium, poses a significant increase in the POAG patients, which could induce PGE2 synthesis to regulate immune responses and elicited proinflammatory cytokine and nitric oxide. (Lamping et al., 1998; Gong et al., 2020) Gut microbiota is typically dominated by the Firmicutes and Bacteroidetes phyla, and it provides key functions for maintaining health, including the production of metabolites that promote homeostasis and competitive exclusion of pathogens. Dysbiosis has been reported in several metabolic disorders and it reconfigures the microbiota, resulting in an increase in the F/B ratio. (Ley et al., 2006; Shin et al., 2015; Yang et al., 2015) In ocular diseases, a high F/B ratio has been observed in dry eye disease, experimental autoimmune uveitis, and age-related macular degeneration mouse models. (Andriessen et al., 2016; Janowitz et al., 2019; Yoon et al., 2021) Consistent with previous studies, we also observed an increased F/B ratio in the intestinal flora of animals with glaucoma, which is a neurodegenerative disease, and F/B ratio was negatively correlated with the number of RGCs.

At the genus level, Akkermansia, Romboutsia, and Bacteroides were the most dramatically increased in the glaucomatous group, whereas Lactobacillus showed the biggest decrease compared with the control group. Previous studies have suggested that these elevated bacteria in glaucoma were all mucus degraders, which were linked to negative health consequences. (Glover et al., 2022) Akkermansia and Bacteroides degraded the mucus when mice are on a diet lacking fiber and the decreased mucus layer was correlated with increased susceptibility to Citrobacter rodentium infection, and also enhanced the growth of the gut pathogens Clostridioides difficile and Salmonella typhimurium. (Ng et al., 2013; Engevik et al., 2021) Akkermansia was also elevated in infectious models of Salmonella Typhi, Salmonella typhimurium, Citrobacter rodentium, and rotavirus infection, as well as in graft-versus-host disease. (Shono et al., 2016; Cannon et al., 2020; Engevik et al., 2020; Hao et al., 2020; Wang et al., 2021) Akkermanisa was also reported to be associated with fecal microbiota transplantation treatment failure in a mouse model of colitis, and elevated Akkermansia (after intake of simple sugars) caused increased susceptibility to colitis. (Ihekweazu et al., 2019; Khan et al., 2020) It is possible that mucus-degradation in glaucoma promotes pathobiont driven inflammation and worsens outcomes. In animal models with alleviated oxidative stress, the levels of these genera are reduced significantly. (Ge et al., 2020; Ruan et al., 2021) In addition, members of the Lactobacillus genus are gram-positive bacteria, and they are used as probiotics or friendly bacteria. Several inflammatory molecules, such as tumor necrosis factor-alpha (TNF-α), nitric oxide, and vascular endothelial growth factor, are upregulated in glaucoma, and they can affect the optic axon. (Vohra et al., 2013) TNF-α receptor 1 activity leads to the recruitment of immune cells, causing the inflammation, oligodendrocyte loss, and activation of enzymes that induce oxidative stress and subsequent RGC death. (Sacca et al., 2019; Ko et al., 2020) Lactobacilli can ameliorate chronic inflammation by decreasing TNF-α expression and may help ameliorate neurodegenerative disease. (Quigley, 2017) We also found that inflammation-related genera, Romboutsia and Bacteroides, are negatively associated with RGCs in a rat model. Research on the pathogenesis of glaucoma is increasingly implicating the inflammatory response and oxidative stress in the pathogenesis of glaucoma. Decreased antioxidant defense or increased oxidative stress may have critical roles in the pathogenesis of glaucoma. (Mousa et al., 2015) Abnormal inflammatory findings are observed in glaucoma, supporting the hypothesis of a dysregulation of the inflammatory balance toward a proinflammatory phenotype. (Baudouin et al., 2021) Hence, flora-targeted therapeutic approaches in glaucoma could be considered.

The primary mode by which gut microbiota interact with the host is *via* metabolites, which are small molecules that are produced as intermediates or end products of microbial metabolism. In our study, the composition of the microbial metabolome differed significantly between glaucomatous rats and controls, and 47 metabolites involved in KEGG pathways were differentially expressed. The differentially expressed metabolites were mainly significantly enriched in bile secretion pathways. The changed bile section route suggested that blood lipid metabolism might be linked to glaucoma, as well as the altered cecal flora and metabolites. Primary bile acids are synthesized from cholesterol and conjugated to glycine or taurine in the liver, stored in the gallbladder, and released into the small intestine, where most are deconjugated by intestinal bacteria and structurally modified into secondary bile acids. Inflammation and oxidative stress can cause hepatic and biliary damage, which affects bile secretion. (Karaman et al., 2006; Preidis et al., 2014)

In the current study, the differentially expressed microbiota-derived metabolites enriched in bile secretion pathways included bilirubin, lithocholic acid, deoxycholic acid, quinine, taurocholic acid, prostaglandin F2 alpha, GSH, and spermidine. Lithocholic acid and taurocholic acid, which are secondary bile acids, had large AUCs and were significantly associated with microbial levels. The metabolite levels are influenced by the proportions of bacteria in rat models. A previous study detected a higher level of 3a,7a-dihydroxycholanoic acid (which is a bile acid) in POAG patients than in controls. (Gong et al., 2020) Parkinson’s disease is accompanied by an increase in plasma levels of cholic acid, deoxycholic acid, and lithocholic acid. (Yakhine-Diop et al., 2020) Oxidative stress is thought to trigger liver injury and play a causative role in hepatic fibrosis development. Lithocholic acid accompanied by reactive oxygen species may induce liver injury and cirrhosis. (Masubuchi et al., 2016) Taurocholic acid is a potent liver metabolite that impairs platelet function and promotes fibrinolysis through calcium regulation. Taurocholic acid has the potential in impaired hemostasis and containment of bile, in addition to causing systemic impairment of coagulation. (Wiener et al., 2015) However, the effects of increases in lithocholic acid and taurocholic acid in glaucoma require further investigation.

GSH is a key component of redox homeostasis, and increased oxidative stress alters GSH metabolism. In our study, metabolomics and ROC analysis suggested that GSH may be a metabolic biomarker in glaucoma models. GSH was downregulated in the fecal and blood of glaucomatous rats, and negatively correlated with cecal microbiota, and positively correlated with the number of RGCs. POAG and normal tension glaucoma patients also exhibit decreased GSH in blood, which is consistent with our results. (Gherghel et al., 2013) The antioxidant function of GSH is mainly achieved by GSH peroxidase-catalyzed reactions, which reduce hydrogen peroxide and lipid peroxide. Oxidized GSH is reduced back to GSH by reductase using NADPH, forming a redox cycle. Many transcription factors and signaling molecules have critical cysteine residues that can be oxidized and this is an important mechanism through which reactive oxygen and nitrogen species regulate protein function and cell signaling that can be modulated by GSH. (Lu, 2009) The high levels of oxidative stress at both the ocular and systemic levels in glaucomatous neurodegeneration are important. (Gherghel et al., 2005; Majsterek et al., 2011) Our results also indicate oxidative stress-related microbial and metabolic alterations in the gut-eye axis, which provide important clues to glaucoma pathogenesis and potential neuroprotective therapies.

It is also important to note that our study has limitations. The use of rat models is a preliminary exploration of the eye-gut axis in glaucoma, and large-scale studies of patients with POAG are needed in the future. In addition, we found that the expression of GSH, an important antioxidant, was decreased, suggesting that oxidative stress may play a role in the eye-gut axis of glaucoma, while the specific mechanism needs to be further explored.

To our knowledge, this is the first study that has focused on the microbial profile and derived metabolites in glaucomatous rats using full-length sequencing and untargeted metabolomic analyses. Increased Firmicutes/Bacteroidetes (F/B) ratio, Verrucomicrobia phylum, and some bacterial genera (Romboutsia, Akkermansia, and Bacteroides) in glaucomatous rats, and enrichment in the bile section pathway suggest a link to oxidative stress, which is consistent with the decreased blood levels of GSH. Therefore, the alternation of gut microbiota and derived metabolites may provide new insights into the pathogenesis of glaucoma, and into restoring a normal microenvironment to treat or prevent glaucoma.

## Data availability statement

The data that support the findings of this study are avilable in Genome Sequence Archive at https://bigd.big.ac.cn/gsa/browse/CRA007159. (Reference number: CRA007159).

## Ethics statement

The animal study was reviewed and approved by the Ethics Committee for Animal Studies at the Eye and ENT Hospital of Fudan University, Shanghai, China.

## Authors contributions

YZ and YL contributed to conception and design of the study. YZ and XZ organized the database and performed the statistical analysis. YZ wrote the first draft of the manuscript. XZ and YL wrote sections of the manuscript. All authors contributed to manuscript revision, read, and approved the submitted version.

## Funding

This research was funded by research grant from the National Natural Science Foundation of China (81970780), and the Shanghai High Myopia Study Group.

## Conflict of interest

The authors declare that the research was conducted in the absence of any commercial or financial relationships that could be construed as a potential conflict of interest.

## Publisher’s note

All claims expressed in this article are solely those of the authors and do not necessarily represent those of their affiliated organizations, or those of the publisher, the editors and the reviewers. Any product that may be evaluated in this article, or claim that may be made by its manufacturer, is not guaranteed or endorsed by the publisher.

## Supplementary material

Supplementary Figure 1 Venn diagram representation of the shared and exclusive OTUs in glaucoma and control groups. OUT, operational taxonomic unit.
