# Supplementary figures and images for: Gut microbiota and derived metabolomic profiling in glaucoma with progressive neurodegeneration
Source: Front Cell Infect Microbiol. 2022 Aug 12;12:968992. doi: 10.3389/fcimb.2022.968992 (PMC9411928; doi:10.3389/fcimb.2022.968992)

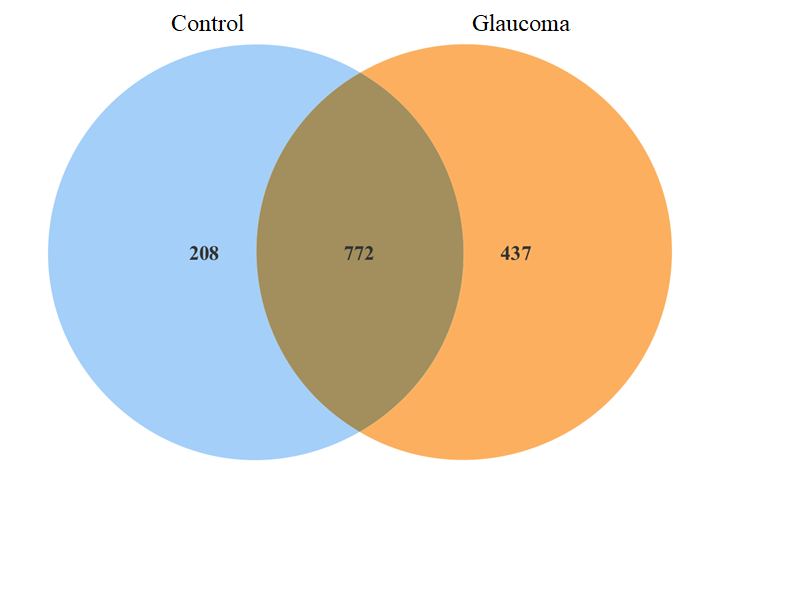

Supplement: Supplementary Figure 1 — Venn diagram representation of the shared and exclusive OTUs in glaucoma and control groups. OUT, operational taxonomic unit. [file Image_1.tif]
