# Supplementary material for: Gut microbiota and derived metabolomic profiling in glaucoma with progressive neurodegeneration
Source: Front Cell Infect Microbiol. 2022 Aug 12;12:968992. doi: 10.3389/fcimb.2022.968992 (PMC9411928; doi:10.3389/fcimb.2022.968992)
Supplement: Supplementary file 2 [file Table_1.docx]

| **Supplementary Table 1. Forty-seven differentially abundant metabolites involved in KEGG pathways across glaucomatous and control rats.** | | | | |
| --- | --- | --- | --- | --- |
| **Name** | **KEGG_ID** | **VIP** | **P value** | **UP/DOWN** |
| Quinine | cpd:C06526 | 1.61 | 0.032 | down |
| Maltotriose | cpd:C01835 | 1.59 | 0.015 | down |
| 2,6-Xylidine | cpd:C11004 | 1.74 | <0.001 | up |
| 3-Hydroxylidocaine | cpd:C16560 | 1.44 | 0.010 | up |
| Neopterin | cpd:C05926 | 1.20 | 0.018 | up |
| Biotin | cpd:C00120 | 1.31 | 0.021 | up |
| Panthenol | cpd:C05944 | 1.40 | 0.004 | up |
| Pyridoxamine | cpd:C00534 | 1.45 | 0.002 | up |
| 5,6-Dimethylbenzimidazole | cpd:C03114 | 1.03 | 0.022 | up |
| Prostaglandin F2alpha | cpd:C00639 | 1.05 | 0.009 | up |
| Maltol | cpd:C11918 | 1.22 | 0.006 | up |
| Trehalose | cpd:C01083 | 1.09 | 0.037 | down |
| Kynurenic acid | cpd:C01717 | 1.05 | 0.011 | down |
| Anthranilic acid | cpd:C00108 | 1.75 | 0.001 | up |
| Phenylacetylglycine | cpd:C05598 | 1.42 | 0.002 | up |
| Creatinine | cpd:C00791 | 1.04 | 0.050 | up |
| Spermidine | cpd:C00315 | 1.31 | 0.006 | down |
| Glutathione | cpd:C00051 | 1.25 | 0.002 | down |
| L-Tryptophan | cpd:C00078 | 1.01 | 0.019 | up |
| Thymidine | cpd:C00214 | 1.38 | 0.008 | up |
| Deoxycytidine | cpd:C00881 | 2.04 | 0.003 | up |
| Cortisone | cpd:C00762 | 1.86 | <0.001 | up |
| Deoxycorticosterone | cpd:C03205 | 1.68 | 0.001 | down |
| Desoxycortone | cpd:C03205 | 1.50 | 0.009 | down |
| delta-Tocopherol | cpd:C14151 | 1.62 | <0.001 | down |
| Homogentisic Acid | cpd:C00544 | 1.38 | 0.002 | up |
| Taurocholic acid | cpd:C05122 | 1.47 | 0.006 | up |
| Oleic acid | cpd:C00712 | 2.03 | <0.001 | up |
| Deoxycholic acid | cpd:C04483 | 1.58 | <0.001 | up |
| Lithocholic Acid | cpd:C03990 | 1.16 | 0.011 | up |
| O-Acetyl-L-carnitine | cpd:C02571 | 1.58 | 0.012 | up |
| Bisphenol A | cpd:C13624 | 2.37 | <0.001 | down |
| Docosahexaenoic acid | cpd:C06429 | 1.23 | 0.004 | down |
| Bilirubin | cpd:C00486 | 1.09 | 0.039 | up |
| Linoleic acid | cpd:C01595 | 1.13 | 0.016 | down |
| Arachidonic acid | cpd:C00219 | 1.29 | 0.011 | down |
| Phenylacetaldehyde | cpd:C00601 | 1.59 | <0.001 | down |
| Hippuric acid | cpd:C01586 | 1.50 | 0.005 | up |
| Orotic acid | cpd:C00295 | 1.56 | 0.002 | up |
| dCMP | cpd:C00239 | 1.22 | 0.048 | up |
| Inosine | cpd:C00294 | 1.51 | 0.006 | up |
| Guanosine | cpd:C00387 | 1.32 | 0.022 | up |
| 2-Hydroxyestradiol | cpd:C05301 | 1.07 | 0.043 | down |
| 17α-Hydroxypregnenolone | cpd:C05138 | 1.12 | 0.018 | up |
| Adrenosterone | cpd:C05285 | 1.27 | 0.020 | up |
| Stearic acid | cpd:C01530 | 1.03 | 0.049 | up |
| Palmitic acid | cpd:C00249 | 1.22 | 0.018 | up |
| KEGG, Kyoto Encyclopedia of Genes and Genomes; VIP, variable importance in the projection. | | | | |
